# Computational screening and in vitro evaluation of sphingosine-1-phosphate analogues as therapeutics for Non-Hodgkin’s lymphoma

**DOI:** 10.1038/s41598-025-25129-7

**Published:** 2025-11-28

**Authors:** Ahmed Abdullah Al Awadh

**Affiliations:** Department of Clinical Laboratory Sciences, Faculty of Applied Medical Sciences, Najran University, 198861441 Najran, Saudi Arabia

**Keywords:** Non-Hodgkin’s lymphoma, S1P analogues, S1PR1, Proliferation, Apoptosis, Necrosis, Biochemistry, Cancer

## Abstract

Non-Hodgkin’s lymphoma (NHL) is a prevalent hematological malignancy that includes a variety of B-cell and T-cell proliferations. The S1P (sphingosine-1-phosphate) pathway, involved in cell survival, proliferation, and migration, plays a critical role in NHL pathogenesis. Targeting S1PR1, the receptor for S1P, may provide a therapeutic strategy for NHL. The primary objective of this study was to identify and evaluate the efficacy of S1P analogues against the S1PR1 receptor through computational methods and experimental validation. The crystal structure of S1PR1 was obtained from the Protein Data Bank, and computational methods, including molecular docking, QSAR modeling, and machine learning techniques, were employed to screen 779 S1P analogues. The most promising compounds were further analyzed through molecular dynamics simulations. In vitro, Raji cells were treated with the potent analogue CHEMBL1540377. MTT assay and colony formation assays were used to evaluate cell viability and proliferation. Additionally, apoptosis and necrosis were assessed by AO/EB staining. Computational studies identified several analogues with high binding affinities to S1PR1, including CHEMBL1540377. Molecular dynamics simulations confirmed the stability and binding of CHEMBL1540377 with S1PR1. In vitro assays demonstrated that CHEMBL1540377 significantly reduced cell viability and inhibited colony formation in Raji cells. AO/EB staining revealed that the compound induced both apoptosis and necrosis in the treated cells. This study identifies CHEMBL1540377 as a potent analogue targeting S1PR1 for NHL therapy. The combination of computational and experimental findings provides a strong foundation for future research and potential clinical application of S1P analogues in treating NHL.

## Introduction

Non-Hodgkin’s lymphoma (NHL), the most prevalent hematological cancer worldwide, encompasses a broad spectrum of B-cell and T-cell proliferative disorders^1^. NHL is distinguished from Hodgkin’s lymphoma by unique clinical features, the absence of Reed-Sternberg cells, and differences in CD15 and CD30 staining patterns on histological examination^2^. Although there are over 40 primary subtypes, the two most common forms are aggressive diffuse large B-cell lymphoma (DLBCL) and indolent follicular lymphoma (FL)^3^. Each subtype is associated with specific risk factors, such as the Epstein-Barr virus (EBV) for Burkitt’s lymphoma and the human T-cell lymphoma virus (HTLV-1) for T-cell lymphoma, as well as distinct driver genetic variants^4^. However, global epidemiological data on NHL subtypes are limited, as population-based cancer registries, including those used in the 2018 GLOBOCAN report, do not differentiate NHL subtypes according to the comprehensive WHO classifications^5^.

The most recent GLOBOCAN data revealed that 509,600 new cases of NHL were diagnosed worldwide in 2018, accounting for 2.8% of all cancer diagnoses. The global age-standardized incidence rates for NHL were 6.7 and 4.7 per 100,000 for men and women, respectively^6^. In high and low/medium human development index countries, the incidence rates were 7.8/100,000 and 4.3/100,000 for men and 5.6/100,000 and 2.9/100,000 for women, respectively^6^. According to the most recent WHO classification, DLBCL accounts for approximately 31% of adult NHL cases in Western countries. In 2018, NHL was estimated to cause 248,700 deaths globally, representing 2.6% of all cancer-related fatalities. The cumulative lifetime risk of dying from NHL was 0.33% for men and 0.21% for women. The average age at death was 76 years, with individuals over 65 years accounting for 78.5% of NHL-related deaths. NHL in people under 65 years is frequently associated with immunosuppression, such as in cases of DLBCL^5^. Men globally face a cumulative lifetime risk of developing NHL that is more than double that of women, with contributing factors including chemical exposure, obesity, HIV infection, tobacco smoking, and alcohol use^7^.

There is a well-established link between sphingosine-1-phosphate (S1P) and NHL, highlighting its role in the survival, proliferation, and dissemination of lymphoma cells^8^. Sphingosine kinase catalyzes the conversion of sphingolipid to S1P, a potent lipid mediator^9^. Through S1P receptors, S1P has been implicated in lymphocyte migration, trafficking, and various lymphoid cancers^10^. S1P lyase (SGPL) catalyzes the irreversible cleavage of S1P into hexadecenal and phosphoethanolamine^11^. Alternatively, S1P can be dephosphorylated back to sphingosine by two isoforms of S1P phosphatase (SGPP1 and SGPP2) and nonspecific lipid phosphate phosphatases. Generally, ceramide promotes apoptosis, growth arrest, or senescence, while S1P favours proliferation and survival—a balance referred to as the “sphingolipid rheostat”^12^. This concept extends to the interplay between ceramide and S1P, encompassing receptor-mediated (autocrine, paracrine, and signal amplification loops) and intracellular target protein-mediated effects of S1P^13^. S1P influences diverse processes, including cellular transformation, migration, angiogenesis, lymph angiogenesis, and epigenetic regulation. Dysregulated S1P signaling, with excessive activity, can contribute to pathological conditions, including cancer^10^.

S1PR1, a chemokine receptor belonging to the G-protein-coupled S1P receptor family, mediates immune cell migration. S1P is produced intracellularly by sphingosine kinase (SPHK) 1/2 and subsequently binds to its receptors (S1PR1–S1PR5) on target cells via autocrine or paracrine signaling. S1PR1 transduces intracellular signals through the ERK, Akt, and Rac pathways, promoting cell migration, survival, and proliferation^13^. The expression of S1PR1 has been observed in cell lines and tissues from NHL, suggesting potential biological roles in this disease. Furthermore, different NHL subtypes exhibit differential expression of S1P receptors (S1PR1-5). Therefore, this study aims to use computational methods to screen and evaluate the efficacy of S1P analogues against S1PR1, the binding protein implicated in NHL^10^.

## Materials and methods

### Protein and compound library

**Fig. 1:**
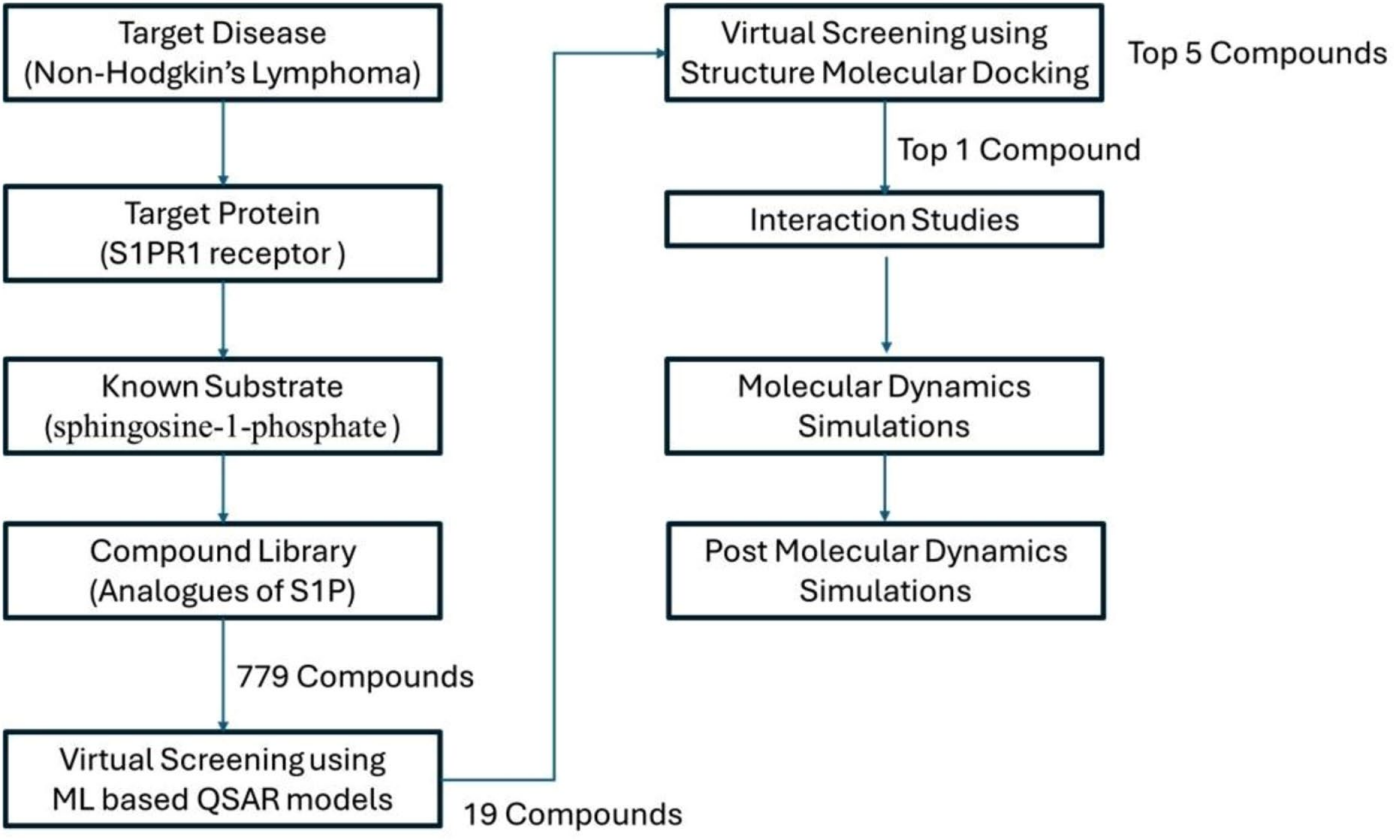
Workflow of the analytical pipeline utilized in the study.

In this study, S1PR1 complexed with uridine-5′-monophosphate (U5P) was selected as the target protein. The crystal structure was obtained from the Protein Data Bank (PDB) using its ID^14^. The homo-dimeric structure of the protein allowed for the extraction of chain A for analysis. Residues within six angstroms of the S1P molecule were used to define the binding site and create the grid box for subsequent molecular docking studies. S1P served as the control substrate in this investigation, and analogues were generated using the Poly Pharmacology Browser 2 (PPB2) server. According to PPB2, molecules most similar to S1P were identified based on their association with the target in the ChEMBL database, yielding a total of 799 analogues. For virtual screening, machine learning (ML) models were employed to screen these analogues. A random forest model proved most effective for selection. The model was trained on the ChEMBL dataset containing analogues with IC50 values. Open Babel was used to convert each compound’s 3D-SDF file into PDBQT format after energy minimization. The pipeline followed for the Analysis during study is presented in Fig. 1**.**

### QSAR modeling

Quantitative structure–activity relationship (QSAR) models were created for virtual screening of natural organic compounds using machine learning techniques. Six regression models were utilized: Bayesian Ridge Regression, Decision Tree Regressor, Support Vector Regression, Linear Regression, Random Forest Regressor, and Gradient Boosting Regression. The ChEMBL database (https://www.ebi.ac.uk/chembl/)^15^was used to obtain the compound library for building the models. The “Target” in ChEMBL with the highest number of associated compounds for NHL was selected, focusing on compounds with IC50 values. IC_50_ values in units of µg/mL were converted to nM (nano-molar) using the equation:

$${IC}_{50} (nM)\frac{{IC}_{50}(\mu \frac{g}{mL})\times {10}^{3})}{Molecular weight (MW)}$$

Following conversion, the log10 of the IC_50_ values was calculated and used for QSAR modeling. Compound properties were computed using RDKit,^16^ and Morgan Fingerprints were applied to develop ML models predicting pIC_50_ based on physicochemical features. The dataset was divided into 70% for training and 30% for testing. Each model’s coefficient of determination (R^2^) was computed to validate performance, with the model showing the highest R^2^ selected for screening. Using this optimized QSAR model, the S1P analogues were screened to approximate bioactivity (IC_50_). Compounds demonstrating higher predicted activity than the control were selected for further evaluation through molecular docking.

### Molecular docking

After selecting the ligands through QSAR modelling, molecular docking analyses was performed to investigate their binding affinities toward the Sphingosine-1-Phosphate Receptor 1 (S1PR1). The three-dimensional structure of human S1PR1 (PDB ID: 7VIE) was retrieved from the Protein Data Bank (https://www.rcsb.org/structure/7VIE). This structure, resolved at 2.9 Å by^14^, represents the receptor in its active conformation complexed with the natural agonist sphingosine-1-phosphate (S1P) and the G < sub > i < /sub > protein. S1PR1 is a member of the G protein-coupled receptor (GPCR) family and plays a crucial role in sphingolipid signaling and immune regulation. For molecular docking analyses, water molecules and heteroatoms were removed, and hydrogen atoms along with Gasteiger charges were added to the target protein^17^. Hundred total runs for each docking experiments were performed for molecular docking to assess the reliability of the molecular docking experiment. The values of 129.5, 142.0, and 108.5 Å in *x*-, *y*, and *z*-axis respectively were set as the grid size for molecular docking studies having grid spacing of 43.4, 85.1, and 47.4 Å. The default values were utilized for the molecular docking analyses parameters through AutoDock 4.2^18^ however the Genetic Algorithm (GA) was employed as a main search protocol^19^. The compounds were ranked based on binding scores and hydrogen bond interactions. The most promising molecules were subjected to dynamics simulation studies.

### Interaction analysis

Protein–ligand interactions were analyzed using the Protein–Ligand Interaction Profiler (PLIP) and Biovia Discovery Studio to visualize 2D and 3D interactions. The selected compounds were further evaluated for ADME (Absorption, Distribution, Metabolism, and Excretion) and toxicity properties to confirm their drug-like characteristics. SwissADME was used to assess ADME properties, while ProTox-II was employed for toxicity predictions.

### Molecular dynamics simulation

Analysing the results from molecular docking analyses and ranking the compound ds based on their binding energies, Molecular Dynamic Simulation analyses was performed for the top ranked compound. Desmond, Schrödinger LLC simulation tool was utilized to heat the selected complex for 303.3 K (K) as an average temperature and MD simulation were performed for 100 ns^20^. The minimization and optimization of the selected complex was performed by employing Protein Preparation Wizard of Maestro^21^. The system was prepared by using the System Builder tool. A solvent model of Transferable Intermolecular Interaction Potential 3 Points (TIP3P) having orthorhombic box was selected^22^. For simulation studies, OPLS 2005 force field was employed^23^. The counter ions were also added in the model to make the model neutral.

Molecular dynamics (MD) simulations were conducted to investigate the dynamic stability and flexibility of protein–ligand interactions. These simulations informed the selection of control ligands and top hit compounds. The Schrödinger suite was used to generate the topology files, hit compound parameters, and standard inhibitor parameter files.

### Trajectory analysis

The Root Mean Square Deviation (RMSD) was calculated to measure the average atomic displacement across all frames relative to the reference frame. RMSD for frame x was calculated using the formula:

$$RMSD_{X} = \sqrt {\frac{1}{N}\mathop \sum \limits_{i = 1}^{N} \left( {r^{\prime}_{i} \left( {t_{x} } \right) - r_{i} \left( {t_{ref} } \right)} \right)^{2} }$$

where *N* is the number of atoms in the selected frame, *t*_*ref*_ is the reference time, r and r′ are the positions of the atoms before and after superimposition, and *t*_*x*_ is the time the frame was recorded.

Ligand Root Mean Square Fluctuation (L-RMSF) was employed to characterize the positional changes in the ligand. The L-RMSF was calculated using a similar formula,

$$RMSD_{i} = \sqrt {\frac{1}{T}\mathop \sum \limits_{t = 1}^{N} \left( {r^{\prime}_{i} \left( t \right) - r_{i} \left( {t_{ref} } \right)} \right)^{2} }$$

where T denotes the trajectory time, *t*_*ref*_ is the reference time, and r and r′ represent the positions of atoms before and after superposition.

### MM-GBSA analyses

Molecular Mechanics—Generalized Born Surface Area was performed to evaluate the binding free energy of the protein ligand complex using the simulation trajectory files from Desmond Schrödinger LLC. The binding free energy ∆G Bind was calculated to be -34.57 kcal/mol indicating thermodynamically favourable and stronger interaction between receptor and ligand.

### Cell culture

Raji cells (human Burkitt’s lymphoma cell line) and peripheral blood mononuclear cells (PBMCs) were procured from the American Type Culture Collection (ATCC). The cells were cultured in RPMI-1640 medium with 10% FBS and 1% penicillin–streptomycin at 37 °C in a 5% CO₂ incubator. PBMCs were isolated using Ficoll-Paque density gradient centrifugation. Cells were maintained under standard conditions and sub-cultured as required.

### MTT assay

Raji cells and PBMCs were seeded into 96-well plates at appropriate densities and treated with CHEMBL1540377 (0–50 µM) for 48 h. CHEMBL1540377 was obtained from AKos Consulting & Solutions. Post-treatment, MTT reagent (20 µL, 5 mg/mL) was added and incubated for 4 h. Formazan crystals were dissolved in DMSO, and absorbance was measured at 570 nm. IC₅₀ values were calculated based on cell viability percentages.

### Colony formation assay

Raji cells (500 cells/well) were seeded in 6-well plates and treated with CHEMBL1540377 (0, 3.12, 6.25, and 12.5 µM) for 7–14 days. Every three days the media containing the compound was replaced days. Colonies were fixed with 4% paraformaldehyde, stained with 0.1% crystal violet, and colonies with > 50 cells were counted manually to calculate inhibition rates.

### AO/EB staining

For detection of apoptosis, Raji cells were subjected to treatment with different concentrations of CHEMBL1540377 for 24 h. Post-treatment, staining was done with a mixture of Acridine Orange (100 µg/mL) and Ethidium Bromide (100 µg/mL) for 5 min. Stained cells were then observed under a fluorescence microscope. The viable cells showed green fluorescence, early apoptotic cells showed yellow fluorescence, while late apoptotic and necrotic cells appeared red. The cell viability and apoptosis/necrosis ratio were assessed by quantifying the fluorescence intensities.

## Results

### Crystal Structure and Grid Box Preparation

**Fig. 2:**
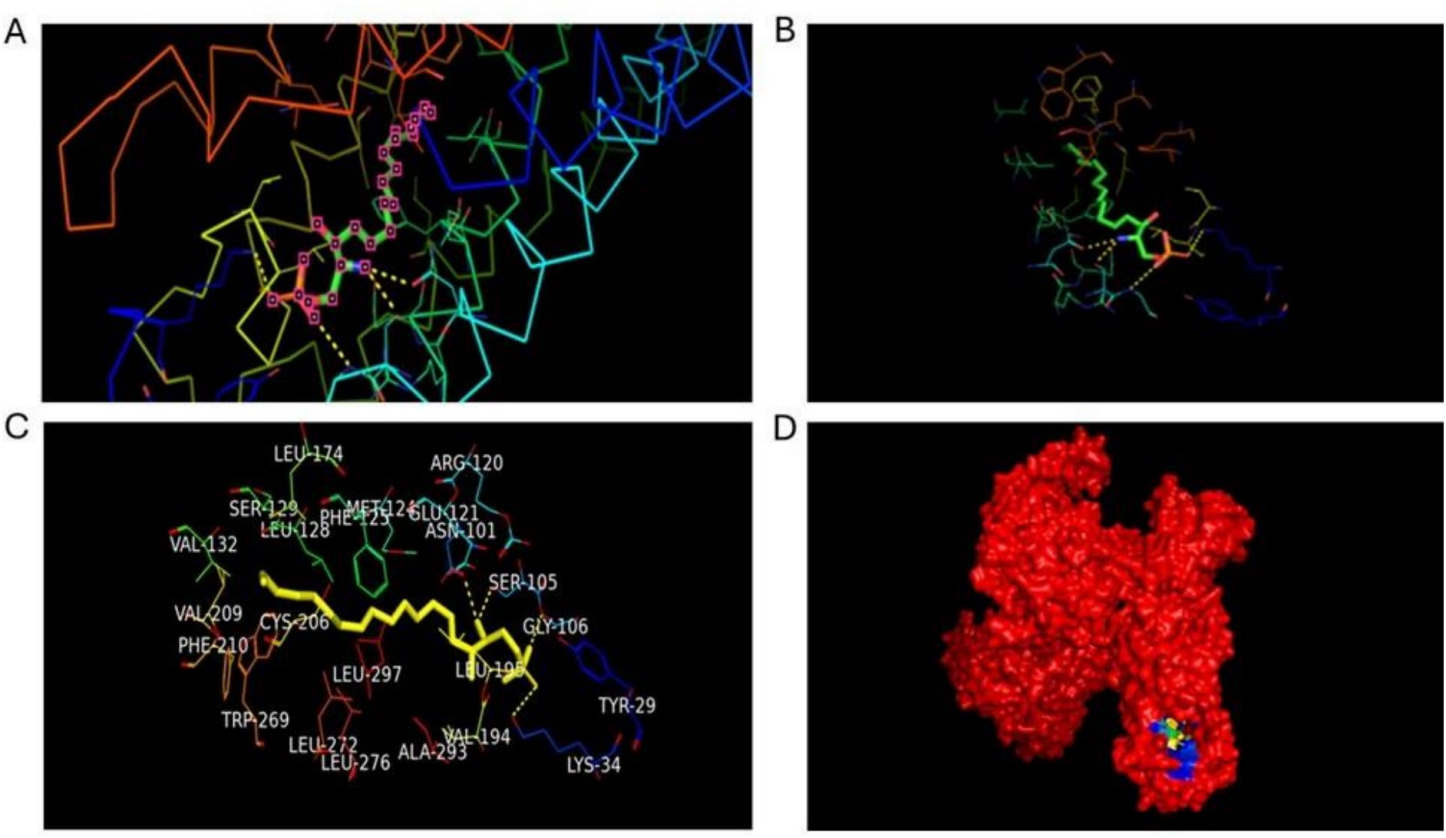
The binding site residues surrounding the S1P ligand on S1PR1 (PDB ID: 7VIE) are shown. (**A**) S1P positioned within the binding pocket of S1PR1; (**B**) the ribbon structure of the protein backbone with S1P attached; (**C**) detailed depiction of binding site residues surrounding S1P; and (D) the entire protein structure with the ligand-binding region highlighted.

The PDB ID 7VIE was used to obtain the S1P from NHL, which is complexed with S1PR1. The resolution of the 3D predicted structure of PDB id 7VIE is 2.86 Å. In this instance, S1P serves as the substrate and is regarded as a control in the investigation, which also included simulation procedures for molecular docking and molecular dynamics. Consequently, the surrounding residues of the S1P coupled to S1PR1 in the experimental structure were included in the grid box for molecular docking (Fig. 2).

### Analogues of S1P

**Fig. 3:**
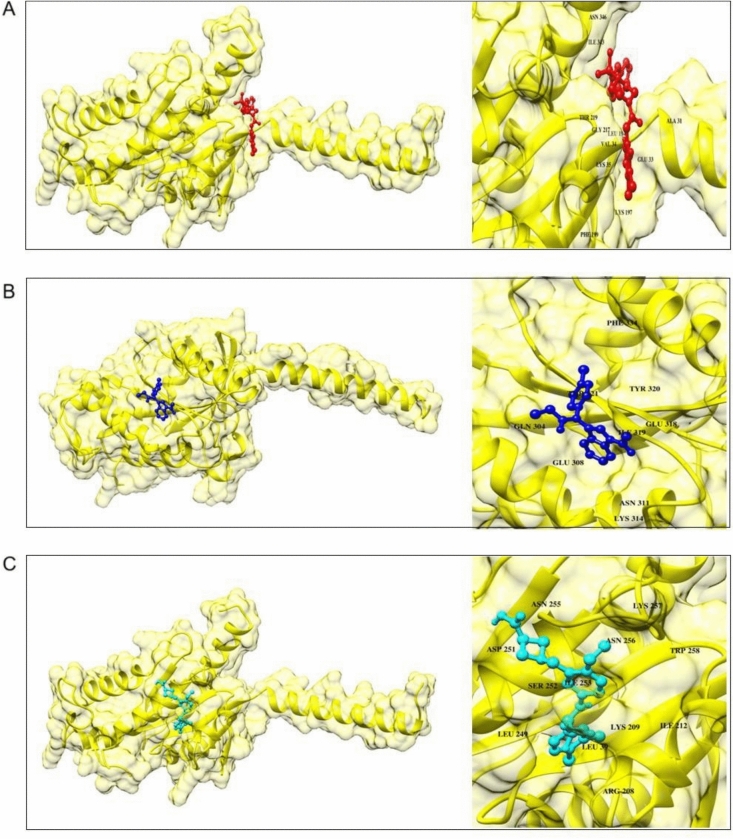
Molecular docking results display the interaction of S1PR1 with selected analogues. (**A**) Docking pose of CHEMBL 1540377; (**B**) docking of CHEMBL 1385784; and (**C**) docking of CHEMBL 3671046. These illustrate the binding conformations and interactions within the receptor pocket.

In this case, analogues of the compound were produced using the substrate S1P, and they were screened against the protein target. Three different approaches were employed to generate analogues of uridine-5′-monophosphate utilizing Poly Pharmacology Browser 2 (PPB2). Using the SMILES representation of S1P as input, PPB2 identified targets with their closest neighbours (Fig. 3).

**Fig. 4:**
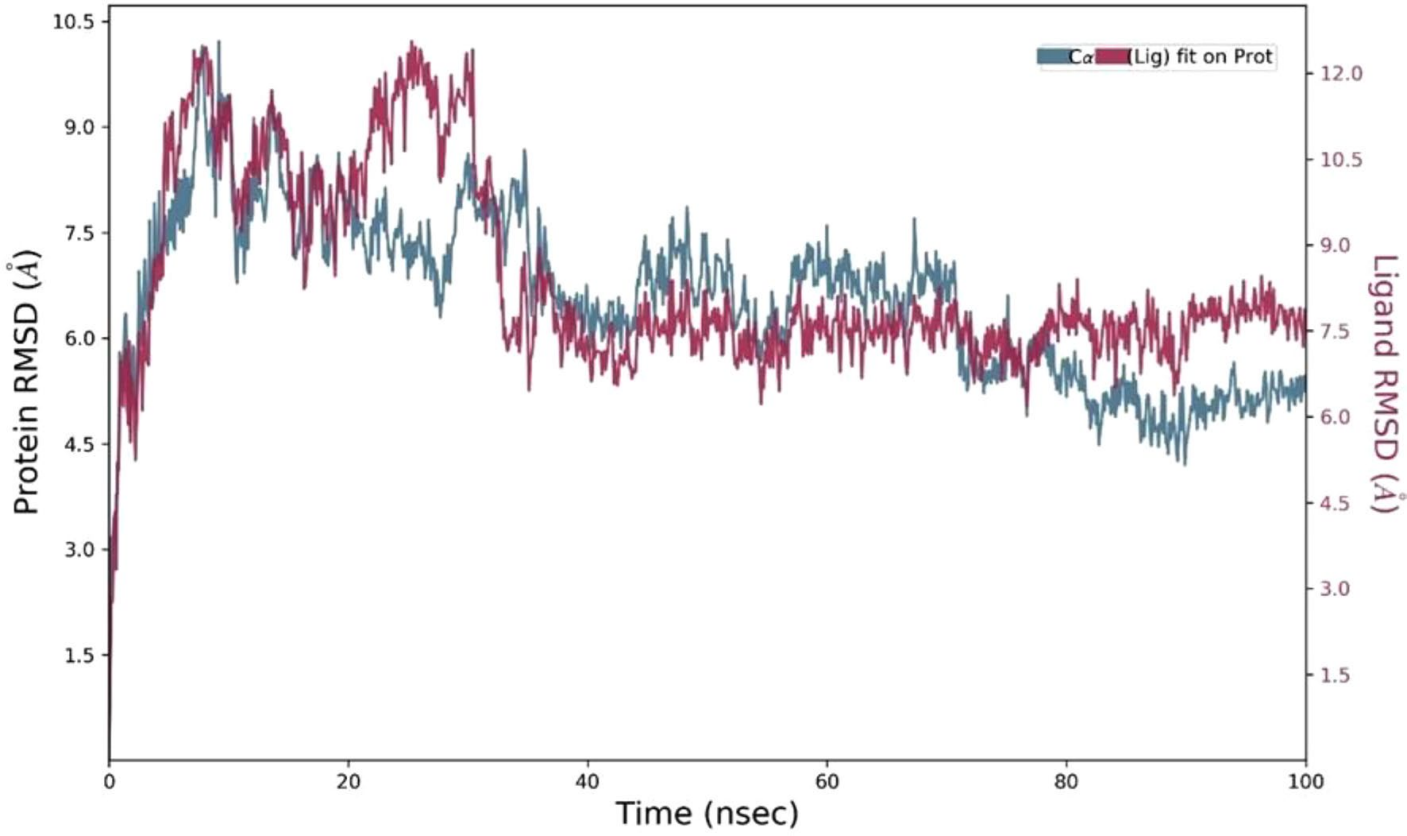
The Root Mean Square Deviation (RMSD) plot demonstrates the stability of the protein-ligand complex over time. The Y-axis on the left shows protein RMSD values, while the right Y-axis indicates ligand RMSD values. The X-axis represents time in nanoseconds.

**Fig. 5:**
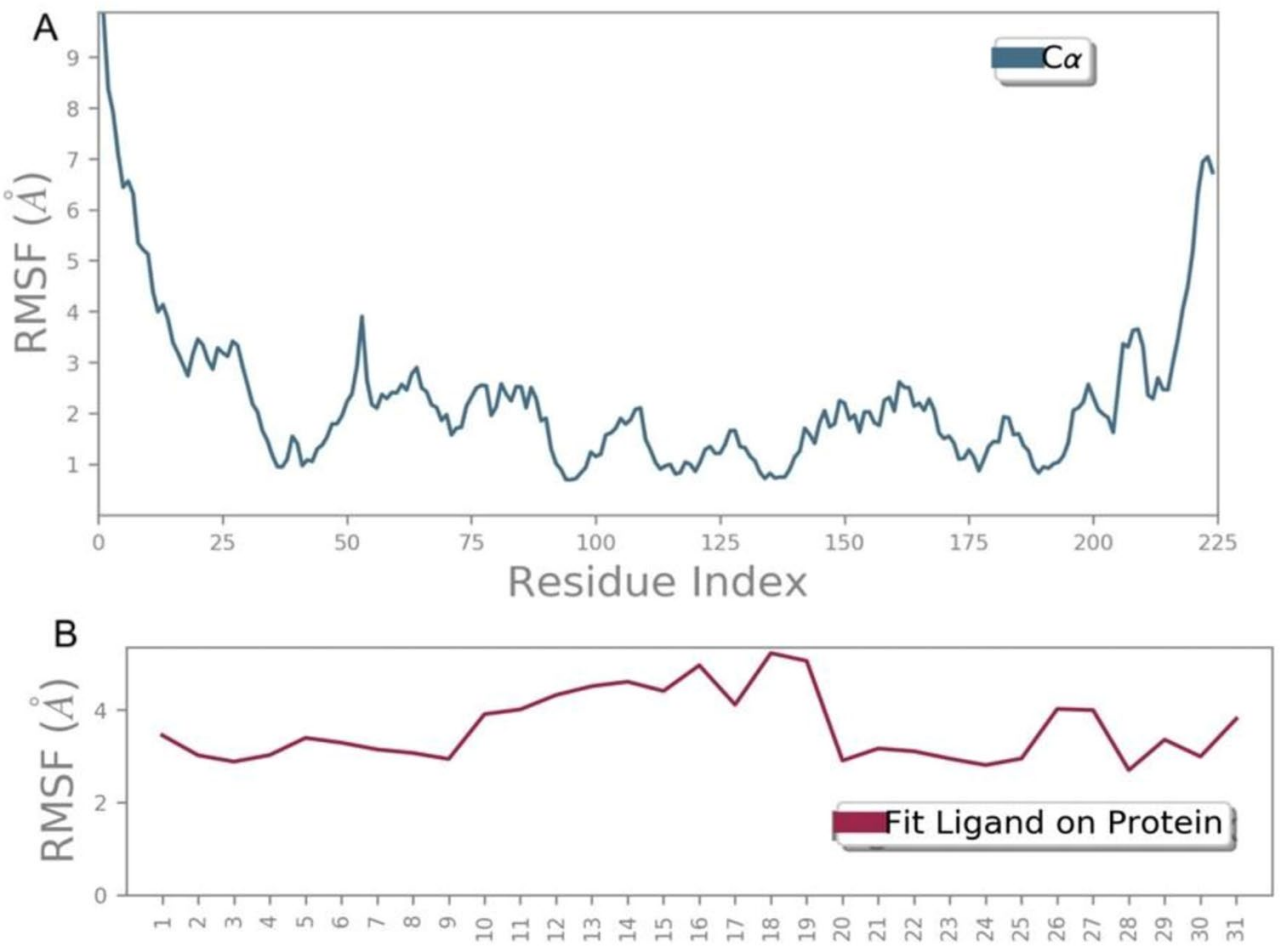
Root Mean Square Fluctuation (RMSF) analysis reveals the stability and flexibility of the protein-ligand complex. (**A**) RMSF values for the protein chain, and (**B**) fluctuations of ligand atoms during molecular dynamics simulations.

**Fig. 6:**
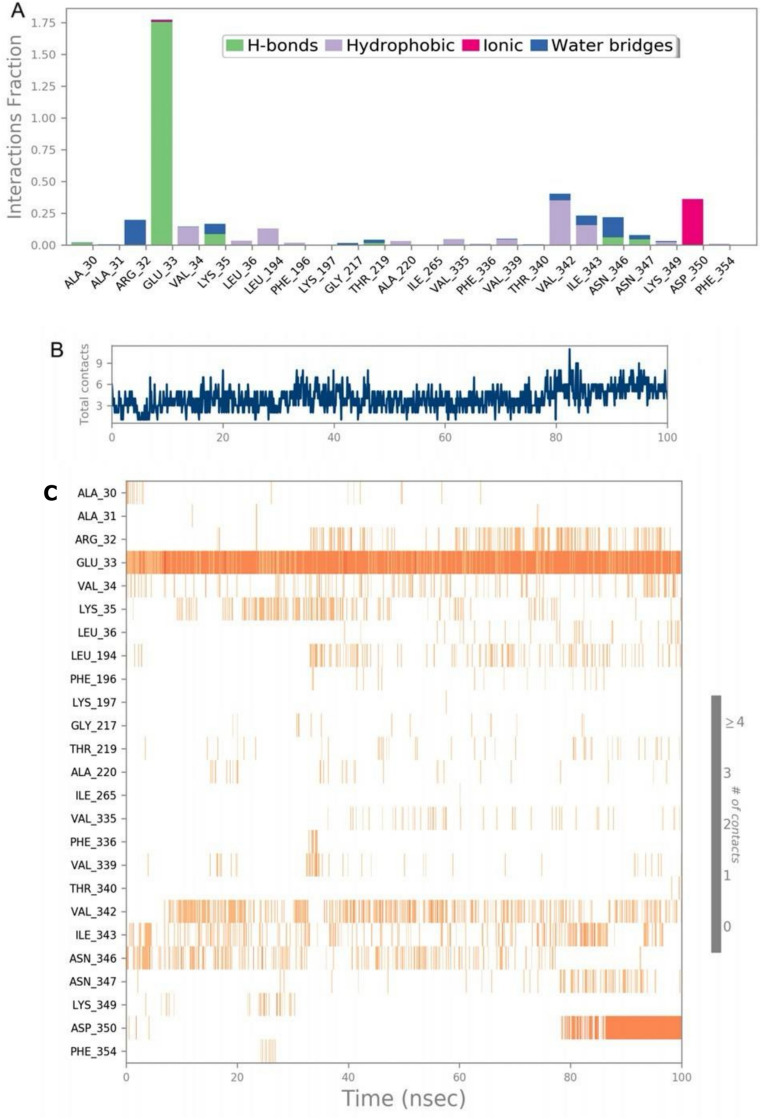
Protein-ligand interactions are analyzed over a 100-nanosecond simulation. (**A**) Types of interactions, including hydrogen bonds, hydrophobic contacts, ionic interactions, and water bridges; (**B**) the total number of contacts between protein and ligand over time; and (**C**) residue-wise interaction profile, where darker shades indicate stronger bonding interactions.

**Fig. 7:**
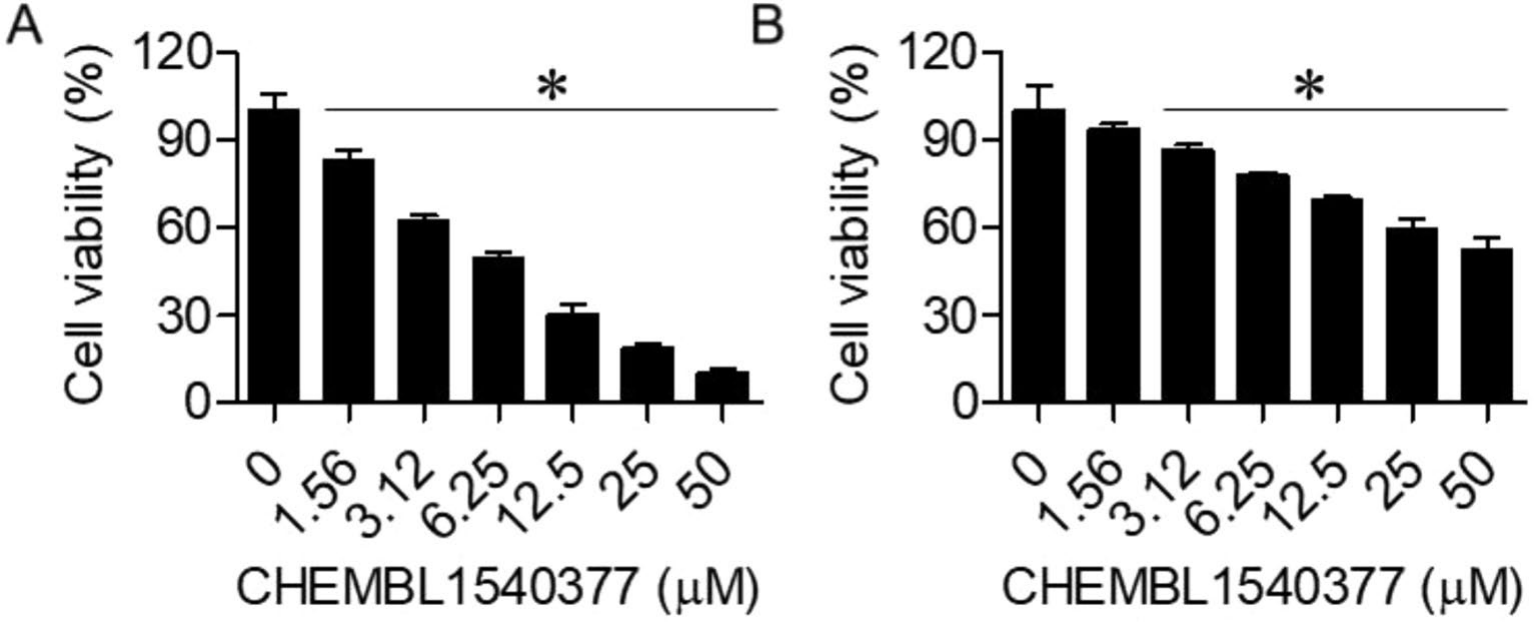
MTT assay results highlight the effects of CHEMBL 1540377 on cell viability. (**A**) Viability of Raji cells and (**B**) viability of PBMCs. The experiments were performed in triplicate, and data are presented as mean ± SD (*P < 0.05).

**Fig. 8:**
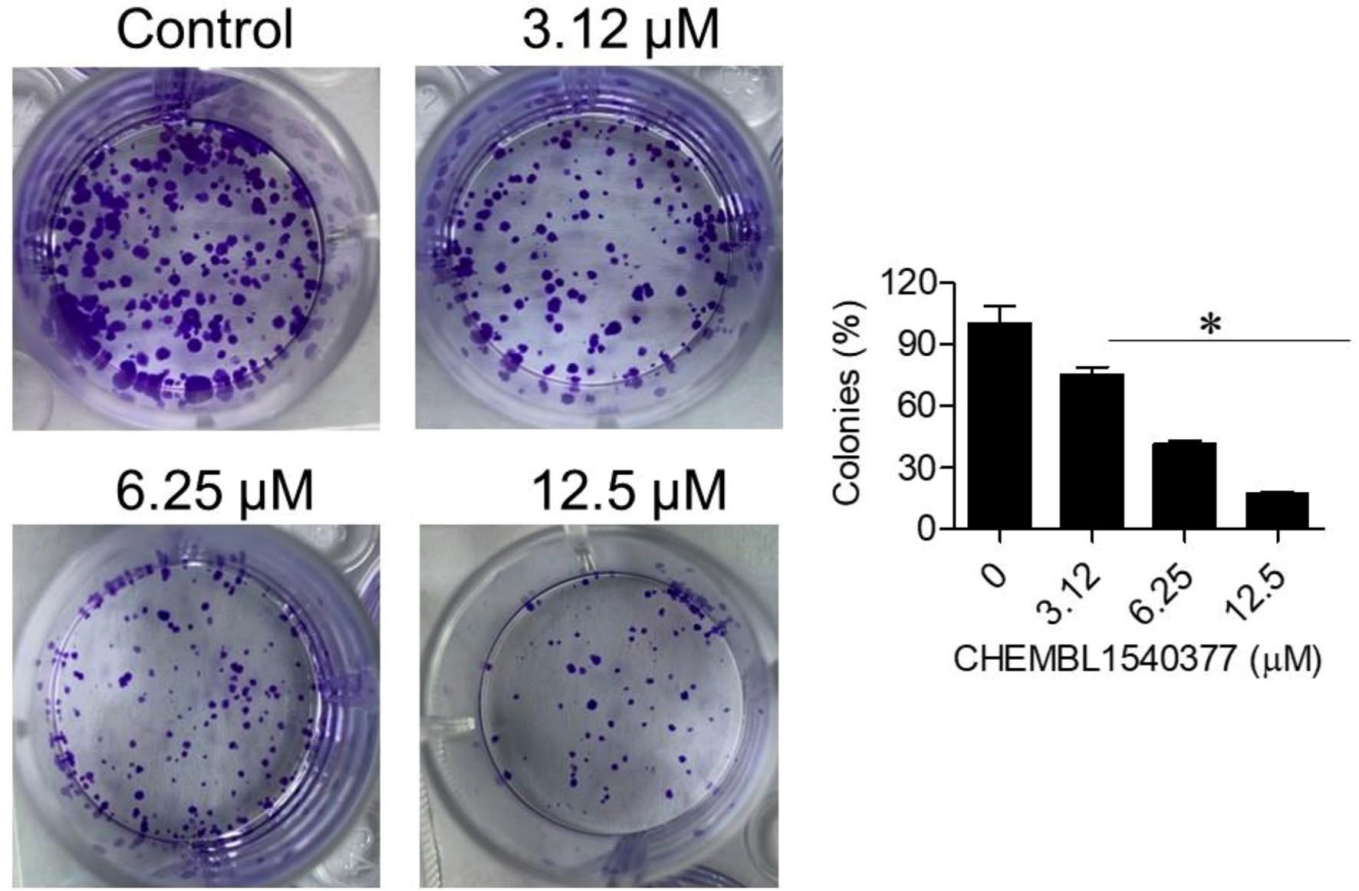
The colony formation assay demonstrates the inhibitory effect of CHEMBL 1540377 on the colony-forming ability of Raji cells. The results are shown as mean ± SD (*P < 0.05).

After determining which molecules in ChEMBL were most similar to S1P, PPB2 selected those compounds to proceed with additional processing. Analogues were selected using S1P as a reference ligand from the CHEMBL dataset via PPB2. A total of 779 analogues were found (Fig. 4). These analogues were employed in the initial screening stage of (Fig. 5) the ML-based QSAR model, as the random forest model proved to be the best for selecting or screening analogues (Fig. 6). The model was first trained on the ChEMBL dataset on analogues having IC_50_ values (Fig. 7). A few experiments also demonstrated the Poly Pharmacology Browser 2 server’s effective utilization (Fig. 8).

### ML-based QSAR model development

**Fig. 9:**
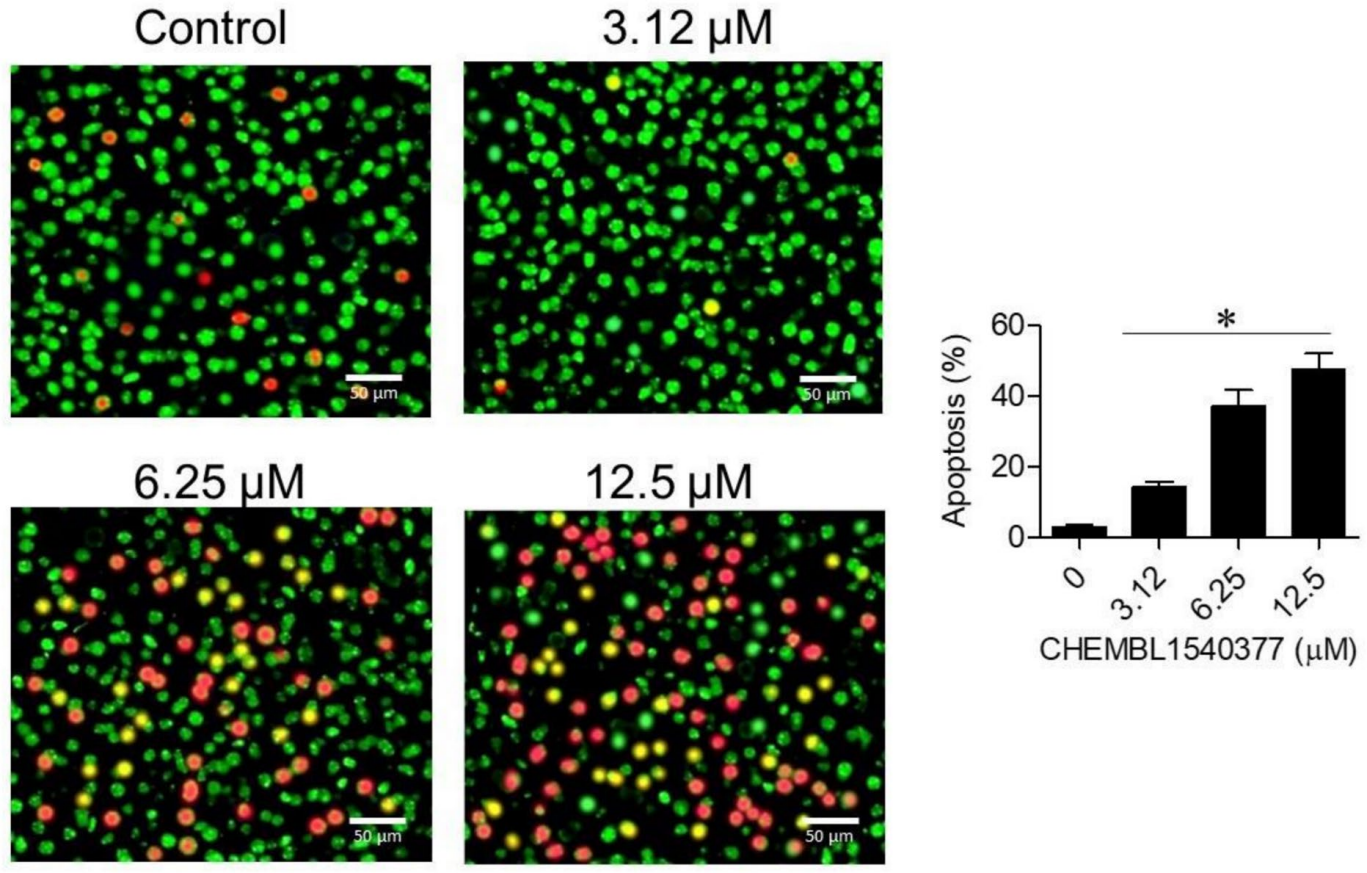
The AO/EB staining assay reveals the pro-apoptotic effects of CHEMBL 1540377 on Raji cells. (A) Apoptosis induced in Raji cells is highlighted, with results presented as mean ± SD (*P < 0.05).

The six regression models—Linear Regression Model, Random Forest Regressor, Bayesian Ridge Regression Model, Decision Tree Regressor, Support Vector Regression Model, and Gradient Boosting Regression Model—were used to perform the ML-based QSAR model. Here, the ChEMBL database was searched to find known inhibitors in order to develop the QSAR model (Fig. 9).

### Molecular docking

**Fig. 10:**
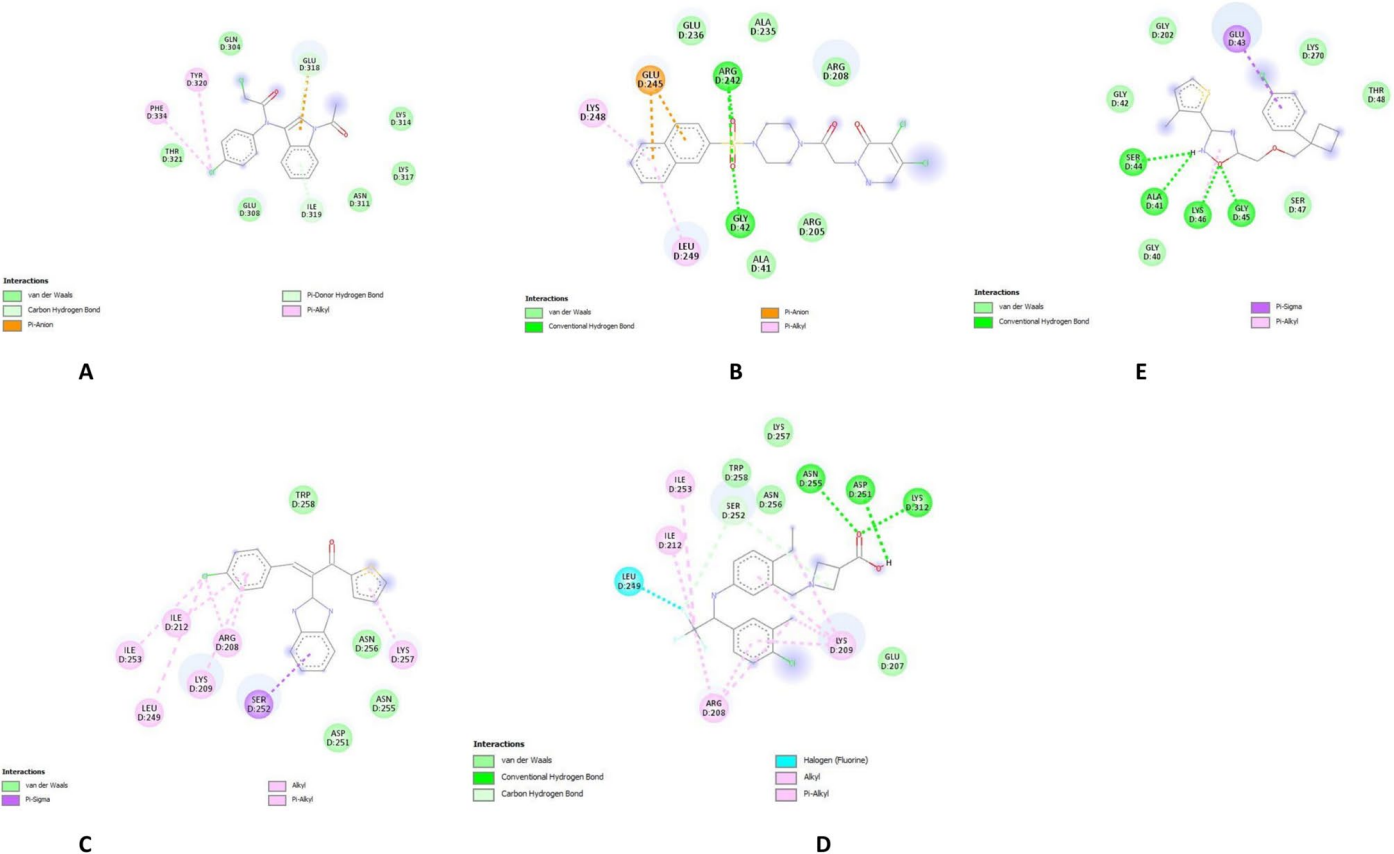
(**a**–**e**) Molecular docking studies 2D interaction.

Molecular docking analysis was performed using the AutoDock Vina tool. To perform molecular docking analysis, the protein was prepared in PyMOL, all the side chains were removed, and the sphingosine was utilized for further processing. Energy minimization of the protein was done using UCSF Chimera. Molecular docking analysis revealed that the ligand CHEMBL1540377 had the maximum binding energy and was utilized for further processing. Binding energies and molecular structures of the first five compounds are given in Table 1. The docking of S1PR1 with the analogue of S1P CHEMBL1540377 is shown in Fig. 3 and Fig. 2D interaction of top potent analogues shown in Fig. 10 a-e.

**Table 1:** Binding Energies and molecular Structures of the shortlisted compounds after docking.

CHEMBL IDs and Ligand Information	Molecular Structure	Binding Energy
CHEMBL1540377 Name: No Data Max Phase: No Data Full Mwt: 481.36 Alogp: 2.24		− 8.1 kcal/mol
CHEMBL1369594 Name: No Data Max Phase: No Data Full Mwt: 364.86 Alogp: 5.70		− 6.3 kcal/mol
CHEMBL1385784 Name: No Data Max Phase: No Data Full Mwt: 361.23 Alogp: 4.86		− 6 kcal/mol
CHEMBL3671046 Name: No Data Max Phase: No Data Full Mwt: 440.89 Alogp: 5.44		− 7.7 kcal/mol
CHEMBL3891659 Name: No Data Max Phase: No Data Full Mwt: 374.89 Alogp: 5.40		− 6.8 kcal/mol

### Trajectory analysis

#### Root mean square deviation (RMSD)

The Root Mean Square Deviation (RMSD) measures the average change in atom displacement for all frames in the reference frame. It is calculated for all frames in the trajectory. The RMSD of the selected ligand CHEMBL1540377 when it was bound to our target protein S1PR1 is shown in Fig. 3.

The results showed high RMSD values in the pre-50-ns phase, which gradually decreased afterward. The binding was stable and did not move away from the target at 100 ns. The plot in Fig. 4 depicts the protein and ligand RMSD values. The plot clearly shows that the difference between the ligand and protein RMSD values is very minor and within the acceptable range. The changes in the ligand and protein are in the range of 1–3 Å. The equilibrated system in the middle and slight convergence at the end indicate stable binding.

#### Root mean square fluctuation (RMSF)

The Root Mean Square Fluctuation (RMSF) is used to measure fluctuations in the protein chains and ligand atoms during simulations. The RMSF of the ligand showed very stable RMSF values between 2 and 4 throughout the simulations, which confirms the stable binding between S1PR1 (target protein) and ligand CHEMBL1540377 (Fig. 5).

### MMGBSA analyses

The vander Waal interaction was found to be -27.38 kcal/mol indicating strong hydrophobic interaction between the binding pocket and ligand during simulation time period. The coulomb energy was found to be -121.6 kcal/mol suggesting some strong electrostatic interactions. The covalent energy was 4.98 kcal/mol suggesting that no covalent bond was formed between the receptor and the ligand throughout the simulation and the Hydrogen bonding was recorded to be -0.41 kcal/mol indicating some hydrogen bonds made during the simulation indicating minimal contribution in the protein ligand interaction. All the calculations mentioned in Table 2.

**Table 2:** Binding free energies between the protein and ligand observed through MMGBSA analyses.

Compound ID	2,585,042
∆G Bind	− 34.57 kcal/mol
vdW	− 27.38 kcal/mol
Coulomb	− 121.6 kcal/mol
Covalent	4.98 kcal/mol
Hbond	− 0.41 kcal/mol
∆GTotal (kcal/mol)	− 178.98 kcal/mol

### Protein–ligand contacts

The stability of the folded protein during ligand–protein interaction is provided by strong hydrogen bonds. To check the stability of the protein–ligand complex, the extent of hydrogen bonds, hydrophobic interactions, ionic interactions, and water bridges were analyzed with different residues of S1PR1 and the selected ligand (Fig. 6A).

The results showed that GLU33 exhibited strong hydrogen bonds with multiple instances of contact with the ligand. When plotted over the timeline of 100 ns (Fig. 6B), average contacts between 2 and 4 were observed. Furthermore, when the residue-wise extent of bonding over the time period of 100 ns was analyzed, GLU33 showed the maximum number of hydrogen bond instances. The residues involved in the interactions included ALA30, ARG32, VAL34, LEU36, LYS197, GLY217, THR219, ILE265, VAL335, and PHE354. The key observations included strong hydrogen bonds with residues GLU33, LYS35, ASN346, and ASN347 and strong hydrophobic interactions with VAL34, LEU194, VAL34, and ILE343 (Fig. 6C).

### Effects of CHEMBL1540377 on Raji Cell Proliferation

The MTT assay revealed that CHEMBL1540377 demonstrated selective cytotoxicity towards Raji cells with an IC₅₀ value of 6.25 µM, while showing minimal toxicity to PBMCs, which exhibited an IC₅₀ value greater than 50 µM (Fig. 7A and B). These results indicate that the compound effectively reduces the viability of cancerous cells without significantly affecting normal cells.

### Effects of CHEMBL1540377 Raji cell Colony formation and apoptosis

The colony formation assay showed that CHEMBL1540377 inhibited the clonogenic potential of Raji cells in a concentration-dependent manner. Untreated control wells produced approximately 120 colonies, while wells treated with 3.12, 6.25, and 12.5 µM of the compound resulted in 90, 50, and 20 colonies, respectively. This corresponds to inhibition rates of 25%, 58%, and 83%, demonstrating the compound’s robust anti-proliferative activity. These findings further corroborate the potential of CHEMBL1540377 as a therapeutic agent against lymphoma cells **(**Fig. 8**).** The AO/EB staining results demonstrated a dose- and time-dependent increase in yellow and red-fluorescent cells, indicating apoptosis, after treatment with CHEMBL1540377. At higher concentrations and longer exposure times, a significant increase in red-stained cells was observed, signifying the induction of late-stage apoptosis (Fig. 9).

## Discussion

Non-Hodgkin’s lymphoma (NHL) is a heterogeneous set of hematological malignancies defined by proliferation of B- and T-lymphocytes^24,25^. The sphingosine-1-phosphate (S1P) signaling pathway and in particular its relationship with the S1P receptor 1 (S1PR1) has important functional importance in the induction of cell survival, proliferation and migration^12,26^. These processes are vital for the progression of NHL, making S1PR1 an attractive therapeutic target^27^. The results of the current study provide useful information for the consideration of targeting S1PR1 by computational screening of S1P analogues.

In the present work, a two-stage strategy was used to assess the effectiveness of S1P analogues in NHL therapy. Determination of the residues of selective importance in the S1PR1 binding site and the establishment of a discriminate QSAR model were at the core of the 779 potential analogues screening. Through molecular docking and subsequent molecular dynamics simulations, several compounds were identified with high binding affinities to S1PR1, with CHEMBL1540377 emerging as the most promising candidate. Our molecular dynamics simulations also validated the resilience of the ligand–receptor complex, in terms of fluctuations of the RMSD and RMSF values, revealing stable interactions throughout the simulation time. These findings are consistent with previous studies that highlighted the role of stable interactions between ligands and S1PR1 in modulating receptor function and influencing cellular responses^28^. Beside this MMGBSA results suggest stable binding between the ligand and the receptor. The strong electrostatic contribution is especially promising if the target site has charged residues.

In order to experimentally confirm computational predictions, in vitro Raji cells (a model human B-cell lymphoma cell line) assays were conducted. MTT assay showed a significant cell viability decrease when treated with CHEMBL1540377, suggesting the cytotoxic effect of CHEMBL1540377. This was also corroborated by the colony formation assay, which showed a significant inhibition of cell proliferation. These two assays supported the evidence regarding the anti-proliferative activity of CHEMBL1540377 in Raji cells and therefore support potential of CHEMBL1540377 as a therapeutic agent for NHL.

In addition, the AO/EB staining assay was used to evaluate the cell death mechanism of CHEMBL1540377. Results showed that the compound significantly caused apoptosis and necrosis of Raji cells, and thereby reinforced its involvement in triggering cell death via multiple mechanisms. This concerted induction of cell death is a promising property for therapeutic agents, since CHEMBL1540377 may circumvent the associated resistance mechanisms that are commonly developed in response to single-agent treatments. These findings align with existing literature, where the induction of apoptosis and necrosis has been shown to enhance the therapeutic efficacy of cancer treatments^29,30^.

Although, the study offers encouraging computational and experimental findings, there are some limitations that need be taken care of in future work. In vitro assays were carried out in a single hit lymphoid cell line (Raji), and the validated compounds would be useful to verify in other NHL cell lines to determine their multicentricity. Another point is that although, AO/EB Staining gave some hints about its mechanism of action, additional investigation, for instance, caspase assays or flow cytometry, will help provide a finer mechanistic description. In addition, the pharmacokinetics and in vivo activity of CHEMBL1540377 must be characterized to completely assess its future as a therapeutic agent for NHL.

## Conclusion

Targeting S1PR1 is a promising strategy for Non-Hodgkin’s lymphoma (NHL) due to its role in cell survival, proliferation, and migration. In this study, computational screening of 779 S1P analogues identified CHEMBL1540377 as a top candidate, with molecular docking, molecular dynamics simulations, and MM-GBSA analyses confirming stable ligand–receptor interactions driven by hydrophobic and electrostatic forces.

Experimental validation in Raji cells demonstrated significant anti-proliferative effects and induction of apoptosis and necrosis, highlighting CHEMBL1540377’s potential to trigger cell death via multiple mechanisms. While further studies across additional NHL cell lines, mechanistic assays, and in vivo evaluations are needed, these findings support CHEMBL1540377 as a promising therapeutic agent for NHL and exemplify the value of integrating computational and experimental approaches in drug discovery.

## Data Availability

The datasets used/or analysed during the current study are available from the corresponding author on reasonable request.

## References

[1] Singh, R. et al. Non-Hodgkin’s lymphoma: A review. *Prim. Care* **9**(4), 1834–1840 (2020). 10.4103/jfmpc.jfmpc_1037_19 PMC7346945 32670927

[2] Parente, P., Zanelli, M., Sanguedolce, F., Mastracci, L. & Graziano, P. Hodgkin Reed–Sternberg-like cells in non-Hodgkin lymphoma. *Diagnostics* **10**(12), 1019 (2020). 33261174 10.3390/diagnostics10121019 PMC7760963

[3] Randall, C. & Fedoriw, Y. Pathology and diagnosis of follicular lymphoma and related entities. *Pathology* **52**(1), 30–39 (2020). 31791624 10.1016/j.pathol.2019.09.010

[4] Kolijn PM, Langerak AW. Immune dysregulation as a leading principle for lymphoma development in diverse immunological backgrounds. Immunol Lett. (2023). 10.1016/j.imlet.2023.08.007 37774986

[5] Mafra, A. et al. Global patterns of non-Hodgkin lymphoma in 2020. *Int. J. Cancer.* **151**(9), 1474–1481 (2022). 35695282 10.1002/ijc.34163

[6] Saputra H, Sriwidyani NP, Paskarani PE, Hartanto H. Challenging case of folliculotropic mycosis fungoides in an 11-year-old girl with erythema cheek plaque: A case report. *Res. Soc. Dev.* (2023).

[7] Thandra, K. C. et al. Epidemiology of non-Hodgkin’s lymphoma. *Med. Sci.* **9**(1), 5 (2021). 10.3390/medsci9010005 PMC7930980 33573146

[8] Wang, Y. et al. FAM19A5/S1PR1 signaling pathway regulates the viability and proliferation of mantle cell lymphoma. *J. Recept Signal Transduct.* **42**(3), 225–229 (2022). 10.1080/10799893.2021.1895220 33685344

[9] Sukocheva, O. A., Lukina, E., McGowan, E. & Bishayee, A. Sphingolipids as mediators of inflammation and novel therapeutic target in inflammatory bowel disease. *Adv. Protein Chem. Struct. Biol.* **120**, 123–158 (2020). 32085881 10.1016/bs.apcsb.2019.11.003

[10] Baeyens, A. A. & Schwab, S. R. Finding a way out: S1P signaling and immune cell migration. *Annu. Rev. Immunol.* **38**, 759–784 (2020). 32340572 10.1146/annurev-immunol-081519-083952

[11] Wigger, D., Schumacher, F., Schneider-Schaulies, S. & Kleuser, B. Sphingosine 1-phosphate metabolism and insulin signaling. *Cell Signal.* **82**, 109959 (2021). 33631318 10.1016/j.cellsig.2021.109959

[12] Bravo, G. Á., Cedeño, R. R., Casadevall, M. P. & Ramió-Torrentà, L. Sphingosine-1-phosphate (S1P) and S1P signaling pathway modulators, from current insights to future perspectives. *Cells* **11**(13), 2058 (2022). 35805142 10.3390/cells11132058 PMC9265592

[13] Fan, X. et al. Recent advances of the function of sphingosine 1-phosphate (S1P) receptor S1P3. *J Cell Physiol.* **236**(3), 1564–1578 (2021). 33410533 10.1002/jcp.29958

[14] Yu, L. et al. Structural insights into sphingosine-1-phosphate receptor activation. *Proc. Natl. Acad. Sci. U S A.* **19**, 119 (2022). 10.1073/pnas.2117716119 PMC9169846 35412894

[15] Gaulton, A. et al. The ChEMBL database in 2017. *Nucl. Acids Res.* **45**(D1), D945–D954 (2017). 27899562 10.1093/nar/gkw1074 PMC5210557

[16] Landrum, G. *RDKit: Open-source cheminformatics*. (2016).

[17] Morris, G. M. et al. AutoDock4 and AutoDockTools4: Automated docking with selective receptor flexibility. *J. Comput. Chem.* **30**(16), 2785–2791 (2009). 19399780 10.1002/jcc.21256 PMC2760638

[18] Huey, R., & Morris, G. M. *Using AutoDock 4 with AutoDockTools: A tutorial*. *The Scripps Research Institute*, La Jolla. (2008).

[19] Morris, G. M. et al. Automated docking using a Lamarckian genetic algorithm and an empirical binding free energy function. *J. Comput. Chem.* **19**(14), 1639–1662 (1998).

[20] Bowers, K. J., Chow, E., Xu, H., Dror, R. O., Eastwood, M. P., Gregersen, B. A., Klepeis, J. L., Kolossváry, I., Moraes, M. A., Sacerdoti, F. D., Salmon, J. K., Shan, Y., & Shaw, D. E. *Scalable algorithms for molecular dynamics simulations on commodity clusters*. In *Proceedings of the 2006 ACM/IEEE Conference on Supercomputing* (pp. 84–94). IEEE. (2006).

[21] Sastry, G. M., Adzhigirey, M., Day, T., Annabhimoju, R. & Sherman, W. Protein and ligand preparation: Parameters, protocols, and influence on virtual screening enrichments. *J. Comput. Aided Mol. Des.* **27**(3), 221–234 (2013). 23579614 10.1007/s10822-013-9644-8

[22] Jorgensen, W. L., Chandrasekhar, J., Madura, J. D., Impey, R. W. & Klein, M. L. Comparison of simple potential functions for simulating liquid water. *J. Chem. Phys.* **79**(2), 926–935 (1983).

[23] Jorgensen, W. L., Maxwell, D. S. & Tirado-Rives, J. Development and testing of the OPLS all-atom force field on conformational energetics and properties of organic liquids. *J. Am. Chem. Soc.* **118**(45), 11225–11236 (1996).

[24] Luo, J. et al. Etiology of non-Hodgkin lymphoma: A review from epidemiologic studies. *J. Natl. Cancer Cent.* **2**(4), 226–234 (2022). 39036553 10.1016/j.jncc.2022.08.003 PMC11256700

[25] Rubio-Jurado, B. et al. New biomarkers in non-Hodgkin lymphoma and acute leukemias. *Adv. Clin. Chem.* **96**, 19–53 (2020). 32362319 10.1016/bs.acc.2019.11.002

[26] Calise, S. et al. Sphingosine 1-phosphate stimulates proliferation and migration of satellite cells: Role of S1P receptors. *Biochim. Biophys. Acta Mol. Cell. Res.* **1823**(2), 439–450 (2012). 10.1016/j.bbamcr.2011.11.016 22178384

[27] Middle, S. et al. Immunohistochemical analysis indicates that the anatomical location of B-cell non-Hodgkin’s lymphoma is determined by differentially expressed chemokine receptors, sphingosine-1-phosphate receptors and integrins. *Exp. Hematol. Oncol.* **4**, 1 (2015). 25938000 10.1186/s40164-015-0004-3 PMC4416323

[28] Jairajpuri, D. S. et al. Identification of sphingosine kinase-1 inhibitors from bioactive natural products targeting cancer therapy. *ACS Omega* **5**(24), 14720–14729 (2020). 32596609 10.1021/acsomega.0c01511 PMC7315586

[29] Carneiro, B. A. & El-Deiry, W. S. Targeting apoptosis in cancer therapy. *Nat Rev Clin Oncol.* **17**(7), 395–417 (2020). 32203277 10.1038/s41571-020-0341-y PMC8211386

[30] Ghobrial, I. M., Witzig, T. E. & Adjei, A. A. Targeting apoptosis pathways in cancer therapy. *CA Cancer J. Clin.* **55**(3), 178–194 (2005). 15890640 10.3322/canjclin.55.3.178

